# A GPU‐based fast Monte Carlo code that supports proton transport in magnetic field for radiation therapy

**DOI:** 10.1002/acm2.14208

**Published:** 2023-11-21

**Authors:** Shijun Li, Bo Cheng, Yuxin Wang, Xi Pei, Xie George Xu

**Affiliations:** ^1^ School of Nuclear Science and Technology University of Science and Technology of China Hefei China; ^2^ Anhui Wisdom Technology Company Limited Hefei Anhui China; ^3^ Department of Radiation Oncology The First Affiliated Hospital of USTC University of Science and Technology of China Hefei China

**Keywords:** GPU, Monte Carlo, MRI‐guided proton therapy

## Abstract

This paper presents the effort to extend a previously reported code ARCHER, a GPU‐based Monte Carlo (MC) code for coupled photon and electron transport, into protons including the consideration of magnetic fields. The proton transport is modeled using a Class‐II condensed‐history algorithm with continuous slowing‐down approximation. The model includes ionization, multiple scattering, energy straggling, elastic and inelastic nuclear interactions, as well as deflection due to the Lorentz force in magnetic fields. An additional direction change is added for protons at the end of each step in the presence of the magnetic field. Secondary charge particles, except for protons, are terminated depositing kinetic energies locally, whereas secondary neutral particles are ignored. Each proton is transported step by step until its energy drops to below 0.5 MeV or when the proton leaves the phantom. The code is implemented using the compute unified device architecture (CUDA) platform for optimized GPU thread‐level parallelism and efficiency. The code is validated by comparing it against TOPAS. Comparisons of dose distributions between our code and TOPAS for several exposure scenarios, ranging from single square beams in water to patient plan with magnetic fields, show good agreement. The 3D‐gamma pass rate with a 2 mm/2% criterion in the region with dose greater than 10% of the maximum dose is computed to be over 99% for all tested cases. Using a single NVIDIA TITAN V GPU card, the computational time of ARCHER is found to range from 0.82 to 4.54 seconds for 1 × 10^7^ proton histories. Compared to a few hours running on TOPAS, this speed improvement is significant. This work presents, for the first time, the performance of a GPU‐based MC code to simulate proton transportation magnetic fields, demonstrating the feasibility of accurate and efficient dose calculations in potential magnetic resonance imaging (MRI)‐guided proton therapy.

## 1 INTRODUCTION

Among many types of radiation therapy, proton therapy is perhaps best known for its inherent promise and controversy associated with a terminology credited to Sr. Bragg.^1^ Protons are praised for their ranges beyond which healthy tissues can be spared from undesirable radiation doses, but the range also makes proton therapy vulnerable to setup error and anatomical variation. The vulnerability and promise of proton therapy are strong enough to justify the need for a new proton dose engine that can live up to the expectation for accuracy and efficiency because after all, even a small shift in the Bragg peak can result in underdose to the tumor that needs to be controlled and overdose to adjacent healthy tissues. Furthermore, such a promising radiotherapy modality must be coupled with a dose engine that is well‐designed for computational speed to match a modern proton clinic workflow. It is well documented that pencil beam (PB) algorithms are computational efficiency but are unfortunately no longer trustworthy in the presence of tissues heterogeneity.^2^, ^3^, ^4^, ^5^ Monte Carlo (MC) methods, on the other hand, have been justifiably welcome by the proton therapy community, considering the reputation of being the gold standard in radiation dose calculations.^1^ Critics, however, are quick to point out that, physical models implemented in these MC codes are complex and general‐purpose MC codes such as GEANT4,^6^ FLUKA^7^ or TOPAS^8^ are time‐consuming, ruling out the possibility for clinical applications. For years, attempts had been made to accelerate MC methods.

Alternatively, in recent years, computing hardware accelerators such as Graphics Processing Units (GPUs) have demonstrated the potential to circumvent this problem by harvesting unprecedented levels of thread‐level parallelism. Indeed, MC algorithms are parallelizable, thus the time‐consuming tasks can be offloaded to a co‐processor such as GPU and be concurrently executed by thousands of threads.^9^ To this end, a fast dose calculator (FDC) track‐repeating algorithm^10^ and a simplified MC algorithm based on measured depth dose distributions in water^11^ have been reported in the proton MC literature. In addition to these two oversimplified algorithms, GPU‐based full MC simulation code had been reported by several groups.^12^, ^13^, ^14^, ^15^, ^16^ What they all have in common is that instead of stimulating a huge number of elastic Coulomb interactions, the Class II condensed history algorithm^17^ was implemented. The main distinction between these approaches lies in the nuclear interactions. In the work of Jia et al^12^ and Schiavi et al,^18^ all materials are simply treated as water whenever nuclear interactions happened. Although most human soft tissues consist of ^1^H, ^12^C, ^14^N, and ^16^O, according to ICRU Report 44, an important element, ^40^Ca in the bone tissues was unfortunately neglected.^19^ A more accurate model of nuclear interactions was implemented by Tseung et al, which could simulate inelastic collisions with any nucleus, but their programming strategy resulted in a slower simulation speed.^14^ Another interesting method was reported in which the ICRU Report 63 database^20^ was used to obtain double‐differential cross‐sections of nuclear interactions and to compute the emission angle and energy of the secondary particles.^21^ The database highly depends on measurement conditions and should be updated in time with the development of detection technology.

In practice, additional margins are introduced on the location of the Bragg Peak due to uncertainties, thus leading to larger treatment volumes.^22^ Uncertainties can be reduced through Image‐guided radiation therapy, and magnetic resonance imaging (MRI) is a prime candidate for this imaging technique. Compared with other imaging modalities, MRI provides unparalleled soft‐tissues contrast while avoiding additional radiation dose to the patient.^18^

These lead to the concept of MRI‐guided proton therapy (MRiPT), which combines the precision of proton beams with real‐time MRI imaging during treatment. Although the clinical feasibility of MRiRT has not yet been demonstrated, magnetic field induced proton dose effects have been reported.^18^, ^21^, ^23^, ^24^, ^25^ This effect for a 90 MeV proton beam in a homogeneous water phantom without and with an air gap was examined by Raaymakers et al.^23^ They found that in the presence of a 0.5 T magnetic field, the impact of the magnetic field on the dose distribution is very small. But soon Wolf and Bortfeld developed an analytical solution to estimate the deflection of protons and found a large deflection for a 200 MeV proton beam which must be accounted for in dose planning.^21^ The more clinically relevant research was done by Hartman et al.^24^ They used TOPAS to simulate and an inverse optimization method to generate intensity‐modulated proton therapy (IMPT) plans in a 1.5 T magnetic field, then confirmed the dosimetric feasibility of real‐time MRI‐guided proton therapy. Recently, by simplifying physical models, a CPU‐based MC method that can simulate magnetic field effects was developed,^25^ and computational efficiency showed considerable improvement but the overall computational performance was still limited by hardware devices.

Motivated by the latest progress on both MRiPT and GPU‐based MC simulations, this paper proposes a new GPU‐based full MC code that focuses on magnetic field for proton therapy and is integrated into the previously reported software platform called ARCHER.^26^, ^27^ This paper is organized as follows: Section 2 describes the physics model and the GPU implementation, Section 3 presents the validation results, and Section 4 makes a conclusion.

## 2 METHODS

### 2.1 Physics model

ARCHER simulates proton transport in voxelized geometry from computed tomography (CT) scans with kinetic energy up to 300 MeV. The method described by Schneider et al^28^ is applied to convert the Hounsfield Unit (HU) of each voxel into density and elemental composition. There are 13 elements and 24 materials predefined. Protons interact with media through various electromagnetic and nuclear interactions. Due to the large number of elastic Coulomb interactions, the analog event‐by‐event simulation is unfeasible. Therefore, the so‐called class‐II condensed history method with a continuously slowing down approximation is employed. Each proton is tracked step‐by‐step until its energy drops below 0.5 MeV or exits the boundary of the phantom.

#### 2.1.1 Sampling step size

At the beginning of each step, the distance for a discrete interaction is sampled based on cross‐section. To avoid the simulation of a large number of elastic Coulomb interactions, the production of δ‐electron (ionization) above threshold energy $T_{e}^{cut}$ is considered as a discrete interaction, whereas the below $T_{e}^{cut}$ event is treated with continuous energy loss. $T_{e}^{cut}$ is a preset value of 0.1 MeV in ARCHER. For materials with the same elemental composition but different density, the total macroscopic cross‐section of this discrete interaction is almost proportional to density. So the total macroscopic cross‐section normalized by the mass density was pre‐computed and tabulated for all material in ARCHER. Elastic and inelastic nuclear interactions are also treated as discrete interactions. Their microscopic cross‐section was extracted from Geant4 using BetheBloch model, hElasticCHIPS model, and Binary Cascade model respectively, and was tabulated in 600 evenly spaced bins ranging from 0.5 to 300 MeV for all 13 nuclei.^29^

Once the total macroscopic cross‐section is determined, the mean free path λ is then calculated. The total number of mean free paths that a particle travels before reaching the interaction point is sampled using Equation (1), where η is a random number uniformly distributed in the range (0, 1). The distance to the interaction point for a discrete interaction iis calculated using Equation (2).

(1) $$n_{\lambda }=-\log\left(\eta \right)$$

(2) $$d_{i}=\lambda_{i}\;n_{\lambda }$$

The cross‐section calculation is based on the physical properties of the current material. It is necessary to ensure that protons do not enter a new volume without stopping at the boundary. The distance to the next voxel's boundary $d_{vox}$ is also calculated, and the smallest of all these distances is chosen as step size δx:

(3) $$\delta x=\min{\left\{d_{vox},d_{i},d_{max}\right\}}_{i\;=\;1,2,3}$$

where $d_{max}$ is a restriction that limits the step size by not allowing the stopping range of protons to decrease by more than 20% during the step. Some research also restricts the maximal step size to 2 mm,^14^, ^30^ which significantly improves calculation accuracy in large volumes where protons may transport a long distance, especially for protons with low high kinetic energy, which can easily deposit lots of energy in one step. However, in our study, simulations were performed on geometry with about 2 × 2 × 2 mm^3^ voxels so the simple boundary limitation applied provides adequate accuracy. When protons transport in range shifters, usually with high kinetic energy, the change of physical parameters such as the restricted stopping power is slight during one step. So, the step size limitation is longer and set to 10 mm, which is an empirical value. After moving a step, either the proton reaches the voxel boundary or a discrete interaction with the shortest distance is performed.

#### 2.1.2 Electromagnetic process

Accurate modeling of the electromagnetic process is essential since protons primarily deposit energy through this process. The electromagnetic process is modeled with continuous energy loss, energy straggling, δ‐electron production, multiple scattering, and deflection caused by magnetic fields. Due to interactions with electrons, a proton continuously loses energy along the trajectory while traveling through matter. The mean energy $\bar{\Delta E}$ decrease experienced by a proton during a step δx is calculated using the following equation:

(4) $$\delta x=\int_{T_{p}}^{T_{p}-\bar{\Delta E}}\frac{1}{L\left(T\right)}dT$$

where L is the restricted stopping power based on the Bethe‐Bloch formula.^31^ The restricted stopping power normalized by the mass density was extracted from Geant4 BetheBloch model^29^ and tabulated for all materials, making the calculation of the equivalent length in water unnecessary.

The total continuous energy loss of a proton is a stochastic quantity with a distribution described in terms of a straggling function. The straggling distribution of δ‐electron production in ionization with energy larger than the cut‐off energy $T_{e}^{cut}$ is implemented by sampling the kinetic energy. The sub‐threshold straggling distribution is modeled by a Gaussian with mean $\bar{\Delta E}$ from Equation (4) and Bohr's variance.^32^

Elastic Coulomb interactions with second electrons above pre‐set energy are treated as a discrete process, named δ‐electron production. It will only be performed at the end of the step when it provides the shortest step length. An Acceptance‐Rejection method is employed to sample the energy of scattered electrons.^30^ The generated δ‐electrons are terminated with their energy deposited locally for simplicity. For 250 MeV protons, the maximum energy of the δ‐electron generated is about 580 KeV, corresponding to about a 2 mm range in water. Since CT pixel spacing is also approximately 2 mm, neglecting electron transport results in negligible errors in most clinical cases. The direction change of the proton in this process can also be neglected according to the fact that the proton mass is much larger than the electron mass.

Protons traversing through matter experience elastic collisions with atomic nuclei, which lead to many small‐angle deflections. In the Class II condensed history algorithm, the multiple scattering theory is applied to calculate the global effect of deflections at the end of the step. According to the central limit theorem, the global effect is a Gaussian distribution with zero means, and its variance is determined by the formula initially proposed by Rossi and Greisen.^33^

Besides multiple scattering, protons can also be deflected by Lorentz's force. In a magnetic field $\vec{B}$, proton normalized direction $\vec{v}$ is changed by adding deflection $\vec{\Delta v}$.^34^

(5) $$\vec{\Delta v}=\frac{c\delta x}{\sqrt{E_{p}^{2}-m_{p}^{2}}}\;\left(\vec{v}\times \vec{B}\right)$$

where *c* is the speed of light, $E_{p}$ is the proton total energy, and $m_{p}$ is the proton rest mass. This formula assumes that proton energy remains constant during a step, so the step length should not be too large. Thanks to boundary limitation and CT's small resolution, this approximation is acceptable.

#### 2.1.3 Nuclear process

Nuclear processes have a significant influence on dose distributions, primarily for proton beams of higher initial energies. The secondary products from the nuclear process are generally emitted at a large angle with a wide kinetic energy range to produce a broad faint halo of dose around the primary proton PB details, which must be taken into account carefully.

The microscopic cross‐section dataset of the X element is extracted from Geant4.^29^ One can obtain the nuclear interaction probability by computing macroscopic cross‐section Σ as below:

(6) $$\Sigma =\rho N_{A}\frac{w_{X}}{A_{X}}\sigma_{X}\left(T_{p}\right)$$

where *w_X_* is the fractional weight, *A_X_* is the atomic mass, ρ is the density, and *N_A_* is the Avogadro constant. Inelastic nuclear interactions lead to several secondary products, including protons, neutrons, deuterons, alpha particles, heavier fragments, etc. Although the binary cascade and pre‐compound models in Geant4 provide a detailed description of nuclear interactions and show good conformity with experiments, they are too complicated and time‐consuming to simulate. For inelastic nuclear interactions between specific elements and specific proton energy, we previously simulated 100 million times in Geant4 to generate a database of nuclear event probabilities. A Python script was developed to analyze the production of secondary particles, and then form a table, which stores the production probabilities as a function of the produced particles’ energy and direction. For each element, the database covers a proton energy range from 10 MeV to 300 MeV, in steps of 10 MeV.

Elastic interactions will lead to the production of a second proton and recoil nuclei, so there are four quantities (energy and scattering angle of the second proton and recoil nuclei) that need to be computed. Once one of the quantities is assigned, the remaining three can be derived based on relativistic kinematics. In ARCHER, the second proton energy after elastic proton–proton interactions is sampled from a uniform distribution, and after elastic proton‐X interactions is sampled by the same method as inelastic interactions.

The long‐range second protons are transported in the same method, with a significant impact on the PB's lateral tails. Heavy fragments, heavy charged particles as well as electrons are terminated with energy locally deposited due to their range limit. Neutrons are neglected since some research had proven that their contribution to the local dose distribution is less than 0.5%.^35^

### 2.2 Code implementation

ARCHER was developed using the CUDA C framework, which is a general‐purpose parallel computing platform and programming model created by NVIDIA to leverage the parallel processing capabilities of GPUs.^26^, ^27^ This enables software programs to perform calculations using both the CPU and GPU. In this section, we will present the GPU code programming and workflow of ARCHER.

The GPU technology was originally designed to accelerate the creation of images for output to a display device. Now it has been considered a new general‐purpose computational tool to perform the computation tasks traditionally handled by the CPU. There are hundreds or thousands of threads in GPU which can be designed to run concurrently, bringing huge parallel processing capability. The GPU card used in this study is one NVIDIA TITAN V card with 512 streaming processors, 14.9 Teraflops floating point capability, and 12 GB global memory.

**FIGURE 1:**
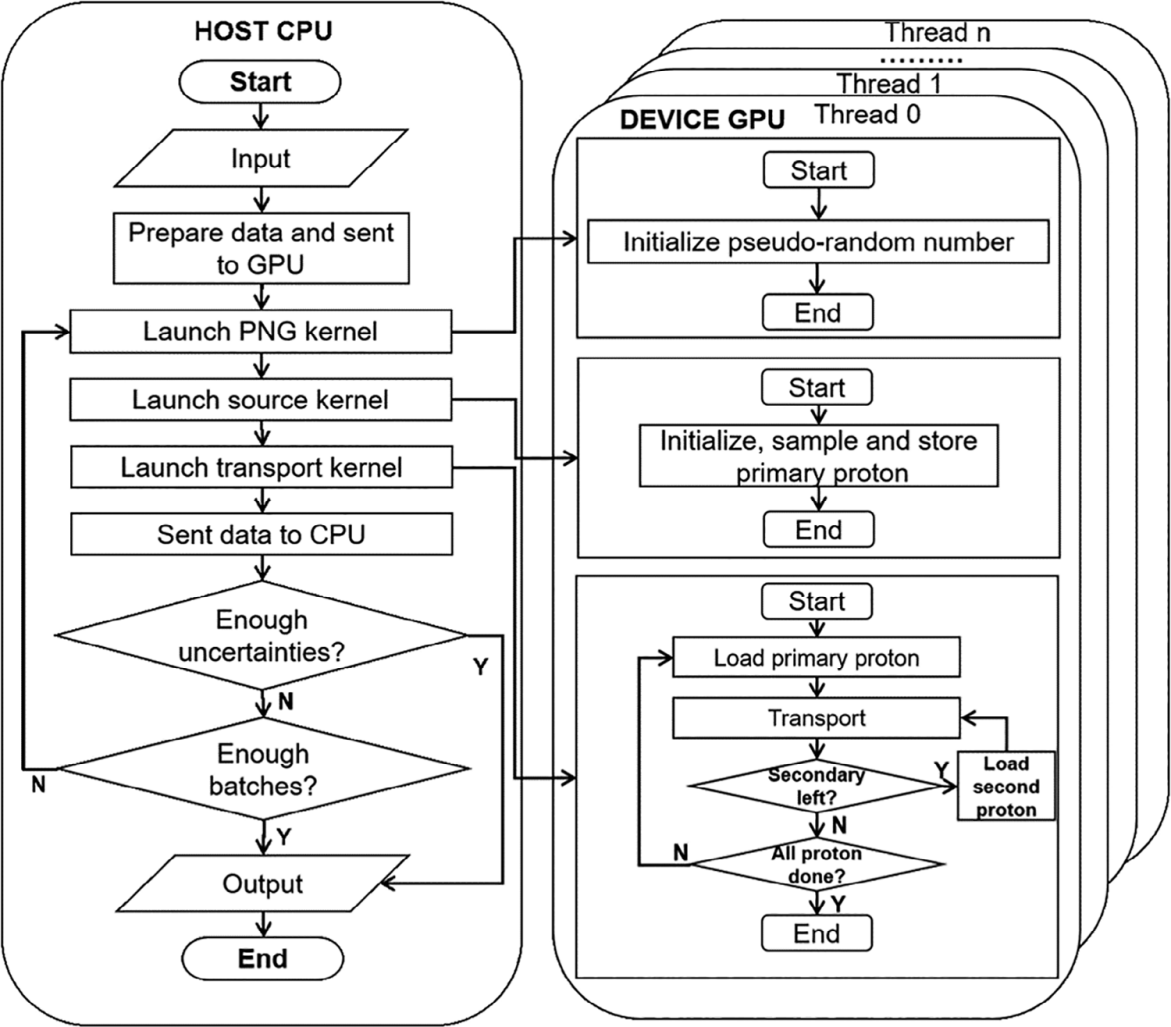
Flowchart of ARCHER MC simulation.

GPU code is composed of two main parts: the host portion, which refers to the CPU and its memory, and the device portion, which refers to the GPU and its memory. The host portion manages memory on both the host and device and launches kernels, which are functions executed on the device. Our code structure follows a typical CUDA execution pattern, as illustrated in Figure 1. Once launched, the host code first reads in all the necessary data, such as voxelized geometry, material properties, and all the cross‐section, then stores them in the host memory. Since kernels can't operate directly on the host memory, device memory must be allocated and data must be copied from the host to the device before executing a kernel. Then the kernel functions are called by the host portion code, which performs them in parallel with a large number of threads on the GPU. Finally, when the kernel call is complete, host and device programs are synchronized and the data are transferred back to the host memory.

Our simulation work is performed in a batched fashion, with three kernels executed in order. Firstly, the PRN kernel initiates a unique independent pseudo‐random number stream for each thread to ensure that pseudo‐random numbers in different threads are not correlated. The CURAND library provided by NVIDIA is employed here, which generates high‐quality pseudo‐random numbers. Next, the source kernel is executed to initiate, sample, and store the primary proton according to the clinical treatment plan. Finally, the transport kernel is launched to record dose depositions. Inside the transport kernel, different threads share the same dose counter‐memory. So, the atomic operation is employed in which multiple threads attempting to increment the dose counter located in one memory location are serialized to ensure a correct result. In each batch, simulation configurations are identical, but the results are different due to random numbers. The corresponding uncertainties are calculated by comparing results from different batches. The more batches we simulate, the smaller the uncertainties. The batch loop is terminated once the uncertainties are less than 1% or after the tenth batch simulation. The dose results are then processed and output after the batch loop.

### 2.3 Testing scenarios

To validate our code, we compared its predictions against the full‐MC code TOPAS in a variety of phantom cases and patient cases. TOPAS is a well‐known MC software that extends the Geant4 Simulation Toolkit to make advanced MC simulation of all forms of radiotherapy easier to use. In our study, we used TOPAS version 3.8, employing a physics list of six models in simulation: g4em‐standard_opt4, g4h‐phy_QGSP_BIC_HP, g4decay, g4ion‐binary cascade, g4h‐elastic_HP, and g4stopping. Other configurations in TOPAS were set to default. For scenarios that do not consider nuclear processes, only the g4em‐standard_opt4 model is used.

Phantom cases simulated here are with dimensions of 10.2*10.2*30 cm^3^ and voxel size of 0.2*0.2*0.1 cm^3^. The benchmarks are performed in four different phantoms: (1) a simple homogeneous water phantom, (2) a homogeneous bone phantom with a density of 1.85 g/cm^3^, (3) a homogeneous tissue phantom with a density of 1.15 g/cm^3^, and (4) a water phantom contained a 5.0 * 5.0 * 5.0 cm^3^ box of bone and a same size box of the lung (0.40 g/cm^3^) which are placed starting at 5 cm depth. To validate the transport in magnetic fields, homogenous magnetic fields with strengths of 0.5, 1.0, 1.5, and 2.0T are applied perpendicular to the beam's direction in water phantoms. In all phantom cases, a mono‐energetic 3*3 cm^2^ proton beam impinges normally on the phantom surface. Ten million primary protons are transported here to make sure the average relative uncertainty is less than 1%.

A total of 15 clinical patient cases from the University of Florida Health Proton Therapy Institute are studied. For each patient case, we also apply 1.5T magnetic fields with direction from feet to head to demonstrate that our code can handle transportation in magnetic fields. The “Emittance” proton beam model described in TOPAS is used. The beam model parameters include spot size sigma, angular variance, and sigma‐angular correlation, which are obtained from our early study.^36^

Comparison metrics adopted in our study are similar to Lysakovski et al.^25^ For Integrated depth‐dose profiles, $\Delta R_{80}$ and relative error $\varepsilon_{rel}$ are calculated.

(7) $$\Delta R_{80}=\left|R_{80}^{1}-R_{80}^{2}\right|$$

(8) $$\varepsilon_{rel}=\;2\frac{\left|d_{1}-d_{2}\right|}{d_{1}+d_{2}}$$

where $R_{80\;}$is defined as the position of the 80% dose in the distal falloff, *d*_1_ is dose of TOPAS, and d_2_ is dose of ARCHER. For magnetic‐field cases, the center of mass (COM) of the beam is introduced.

(9) $$\mathrm{COM}=\frac{1}{N}\sum x_{i}*\;d\left(x_{i}\right)$$

where *N* is the number of voxels, $x_{i}$ is the position and $d(x_{i})$ is the corresponding scored doses. For all cases, we listed a table of gamma pass rate and computing time.

## 3 RESULTS

In this section, we present the simulation results. To demonstrate the accuracy of ARCHER, we compared the dose distribution with TOPAS's results for different geometry and materials, using the comparison metrics mentioned above. Additionally, we demonstrate the efficiency of ARCHER by showing tables of computing time for all cases.

### 3.1 Phantom cases

**FIGURE 2:**
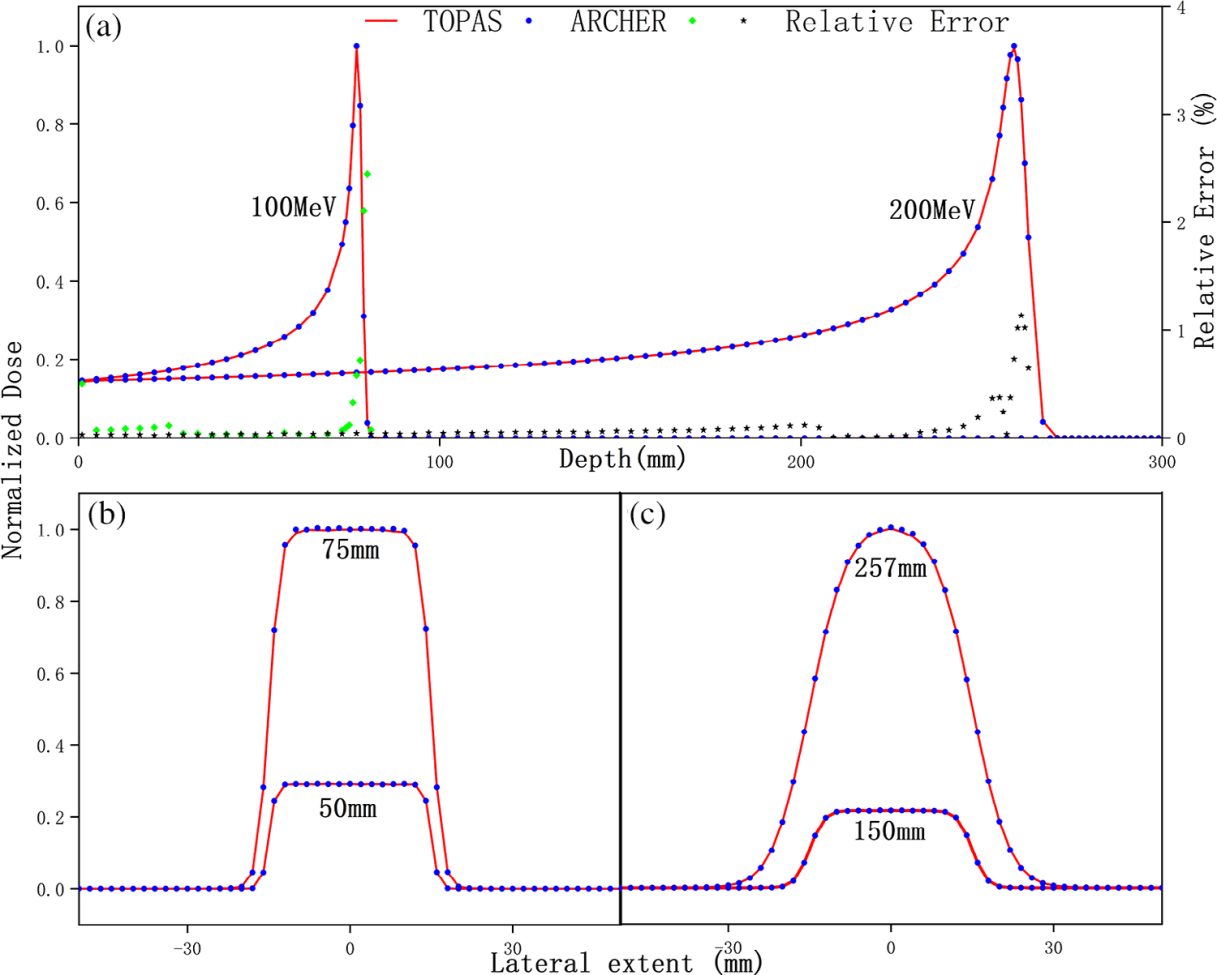
Dose comparison in water only with electromagnetic interactions. In panel (a), integrated depth‐dose profiles are shown. TOPAS simulations are indicated by solid red lines, ARCHER simulations as blue points, and the relative errors are plotted in green and gray dots. In panel (b), two lateral profiles for 100 MeV proton beam are shown. The legend “50 mm” represents the lateral profile obtained at the depth of 50 mm. In panel (c) lateral profiles for the 200MeV proton beam are shown.

In all homogeneous phantom cases, parallel mono‐energetic proton beams with energy of 100 or 200 MeV are studied. Figure 2 shows a comparison of the dose distribution in water when only simulating electromagnetic interactions. The electromagnetic process will be performed in every step, resulting in direction changes and energy decreases, while the nuclear process may not occur during the entire transport. Hence, it is necessary and desirable to verify the accuracy of the electromagnetic process first. In the upper panels (A), integrated depth‐dose profiles of ARCHER and TOPAS are shown. The $\Delta R_{80}\;$are 0.021 and 0.017 mm, and the mean $\varepsilon_{rel}$ for dose greater than 10% of maximum are 0.115% and 0.086% respectively. Lateral dose profiles are shown in panels (B) and panels (C). One of them is obtained in the Bragg Peak region, while the other is in the flat plateau region.

**FIGURE 3:**
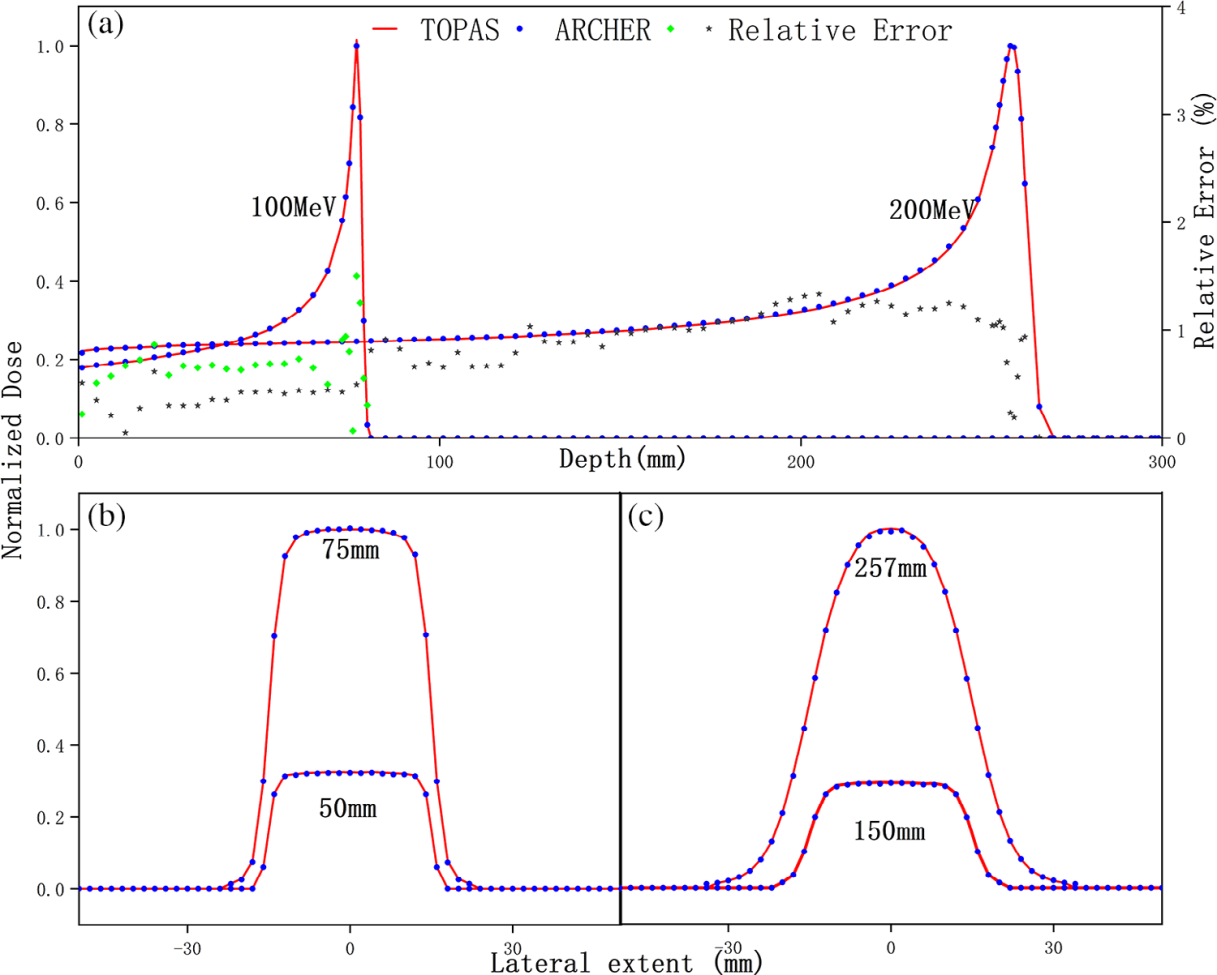
Dose comparison in water with all interactions. All the panels have the same meaning described in Figure 2.

**FIGURE 4:**
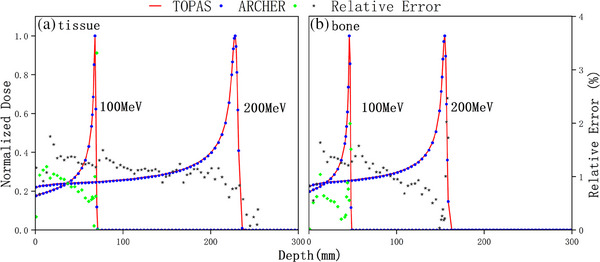
Dose comparison in tissue and bone. Integrated depth‐dose profiles for a tissue phantom and a bone phantom respectively.

Figure 3 illustrates the dose calculation results in water with all interactions. Panels (A) present integrated depth‐dose profiles. We found that TOPAS's results are slightly larger than ARCHER's results when nuclear interactions are activated, especially in the middle‐depth region. This is because neutral particles that arise from inelastic nuclear interaction are neglected in ARCHER. Nonetheless, the dose distribution is still in good agreement with $\Delta R_{80}$ less than 0.055 mm and mean $\varepsilon_{rel}$ less than 0.810%. Lateral dose profiles in water are shown in panels (B) and (C). Figure 4 further demonstrates that the two codes are in excellent agreement with other materials. The mean $\varepsilon_{rel}$ are 0.879% and 1.122% for bone, and 0.841% and 1.123% for tissue.

**FIGURE 5:**
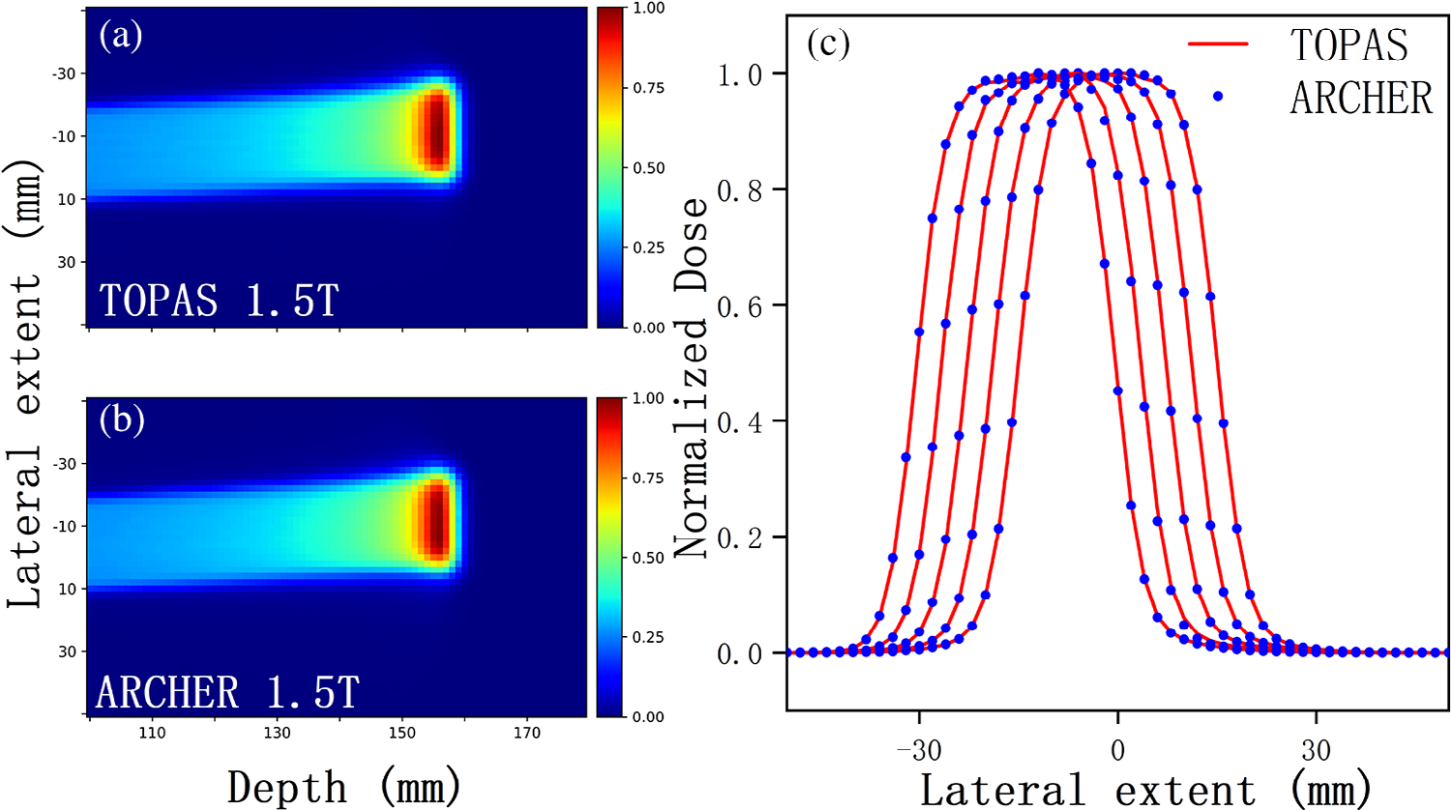
Dose comparison in water with Magnetic field for 150 MeV protons in water, 2D dose distributions calculated with TOPAS (a) and ARCHER (b) are shown in a plane perpendicular to the 2 T magnetic field. In panel C, lateral profiles taken at a depth of 156 mm are shown. Magnetic field strengths are 2T, 1.5T, 1T, 0.5T, and 0T from left to right.

We also add homogenous magnetic fields perpendicular to the beam's direction in a water phantom to validate ARCHER simulation in magnetic fields. Strengths of 0.5, 1.0, 1.5, and 2 T are studied here. A comparison of the dose distribution is shown in Figure 5. In panels (A) and (B), 2D dose distributions for ARCHER and TOPAS, perpendicular to the 2 T magnetic field, are shown respectively. It is evident that proton trajectories are deflected by the Lorentz force, making it necessary to account for this force in dose calculations in the presence of magnetic fields. Panels (C) show lateral profiles at the depth of 15.6 cm for different field strengths. Clearly, lateral profile offset increases with the strength of the field. The difference in COMs stayed below 0.05 mm.

**FIGURE 6:**
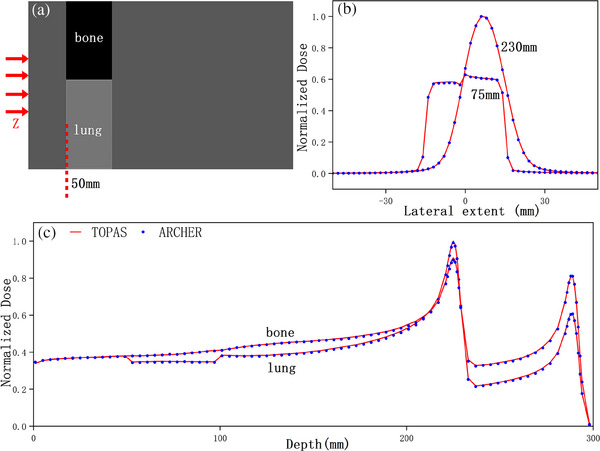
Dose comparison in inhomogeneous phantom. (a) The geometry of inhomogeneous phantom. (b) Lateral profiles at the depths of 75 and 230 mm for 200 MeV protons. (c) Percentage depth dose curves through the bone insert and the lung insert.

To validate ARCHER simulation in heterogeneous media, we study an inhomogeneous phantom. The geometry of the heterogeneous phantom is represented in Figure 6a. Percentage depth dose curves through bone insert and lung insert shown in Figure 6c both have two Bragg peaks due to the different insert materials along the beam path. The mean $\varepsilon_{rel}$of percentage depth dose curves are 1.17% and 0.77% respectively. Lateral dose profiles obtained at the depth of 75 and 280 mm are shown in Figure 6b. In the shallower profile, significant dose changes can be found at the interface of two materials. In the deeper profile, the dose contributions are mostly from the primary proton beam along the lung insert.

Table 1 shows the computation time and 3D γ‐test passing rates between ARCHER and TOPAS. The quantity $\bar{\sigma /D}$ indicates the achieved simulation precision which is defined in Jia et al.^17^ In the 2 mm/2% criterion, the dose percentage threshold is set to 2% and the distance threshold is set to 2 mm. 1 mm/1% criterion is a stricter one which is applied here to further demonstrate the accuracy of ARCHER. During the calculation of the γ‐test pass rate, dose less than 10% of the maximum dose are not considered. In Table 1 we found that γ‐test passing rates of 2 mm/2% are above 99.2%, and even when stricter criterion 1 mm/1% is used, γ‐test passing rates are still high. The computation time ranges from 0.82 to 4.54 s for ARCHER, which can fully demonstrate the high efficiency of ARCHER.

**TABLE 1:** Quantitative analysis table for phantom cases.

Phantom	Energy (MeV)	$\bar{\sigma /D}$ (%)	$P_{\gamma (1\%/1mm)}$ (%)	$P_{\gamma (2\%/2mm)}$ (%)	T_ARCHER_ (s)	T_TOPAS_ (s)
Water EM	100	0.49	99.7	100	0.82	1998
	200	0.54	99.8	100	1.73	6442
Water	100	0.54	98.7	99.9	1.49	2333
	200	0.77	99.5	100	3.59	6822
Water 0T	150	0.63	99.4	99.9	2.42	5462
0.5T	150	0.64	99.4	100	2.79	5451
1T	150	0.65	99.2	99.9	2.74	5443
1.5T	150	0.64	99.1	100	2.73	5485
2T	150	0.64	98.9	100	2.74	5430
Bone	100	0.55	96.5	99.2	1.33	1974
	200	0.74	98.8	99.7	3.18	5399
Tissue	100	0.54	99.4	100	1.51	2984
	200	0.75	99.0	99.9	3.65	7526
Inhomogeneous	200	0.84	99.6	100	4.54	9163

### 3.2 Patient cases

**FIGURE 7:**
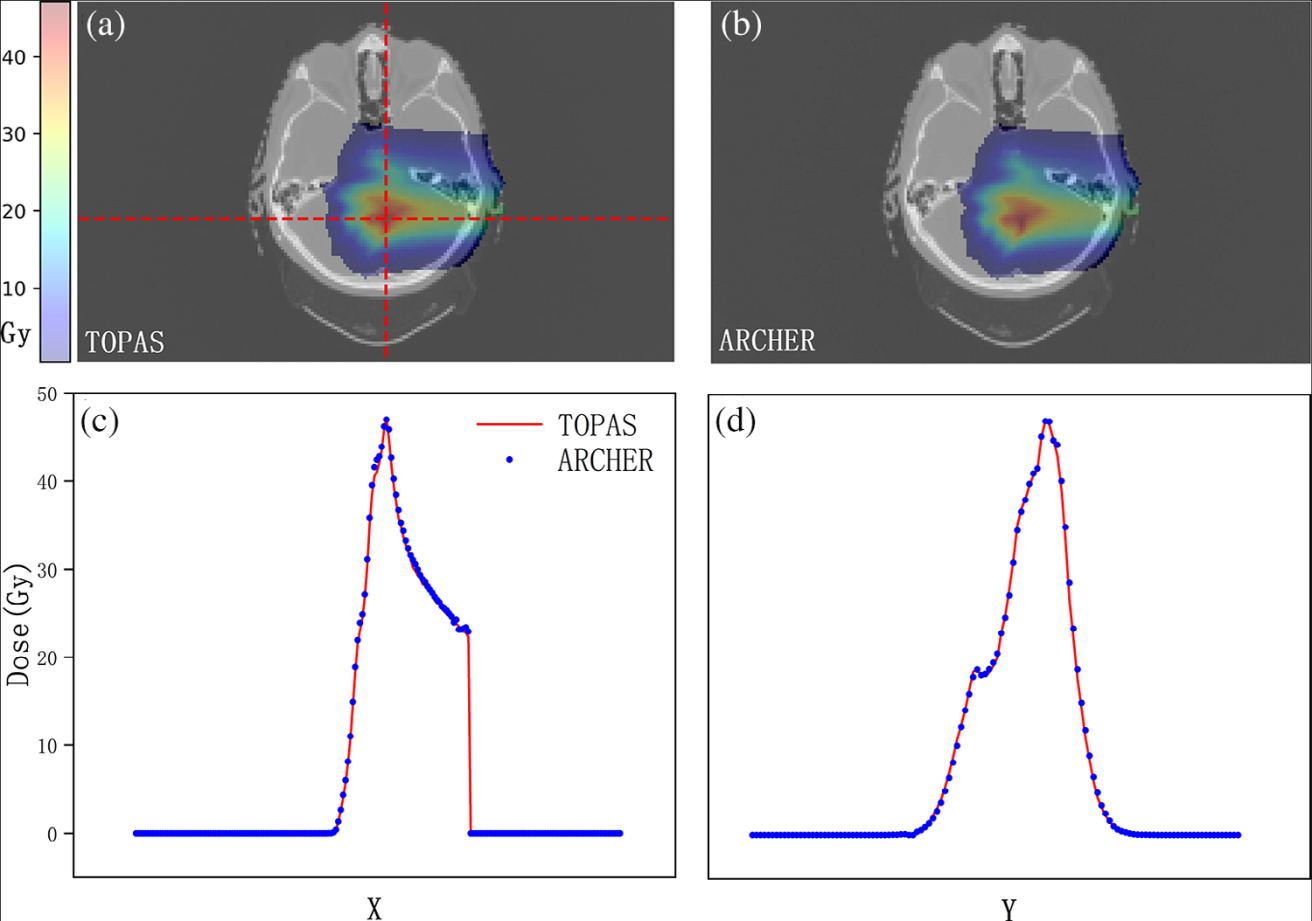
Dose comparison in patient. Axial views of calculated doses for patient cases are shown for (a) TOPAS and (b) ARCHER. Longitudinal and lateral profiles are shown in panels (c) and (d), respectively. The locations of the profiles are indicated with a red dashed line in panel (a).

**FIGURE 8:**
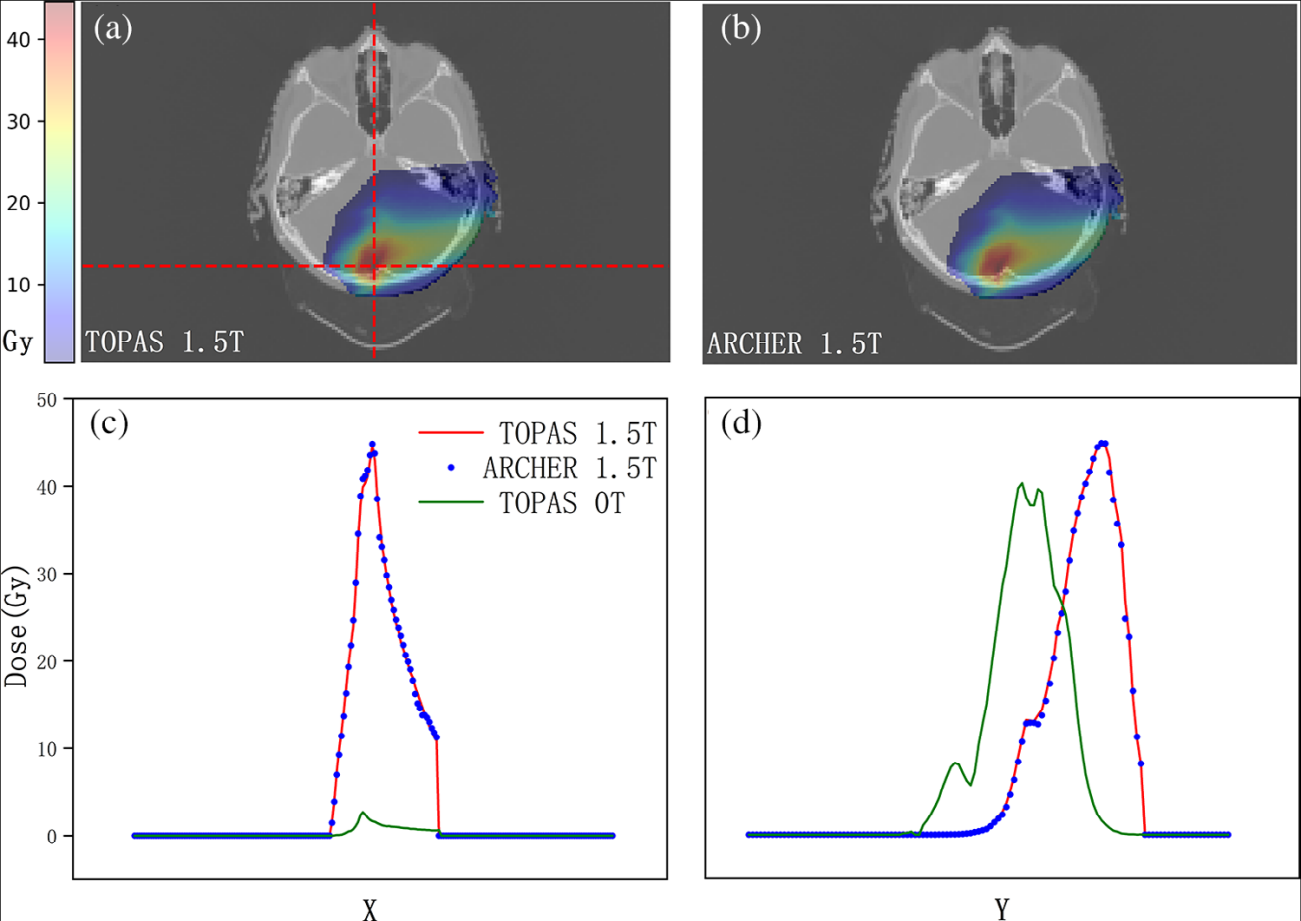
Dose comparison in patient with a magnetic field. The results of the same clinical patient plan with an added perpendicular magnetic field of 1.5 T. All the panels have the same meaning described in Figure 7. Additional profiles of TOPAS without magnetic field are shown in green line in panels (c) and (d).

We performed simulations on 15 clinical patient cases across four clinical sites including head‐and‐neck, breast, lung, and prostate, some of which were treated with range shifters. Beam model parameters are described in our early study,^36^ and the details of delivery were read from the treatment plan. The results are shown in Figure 7. Dose distribution calculated with TOPAS and ARCHER at one transverse slice are shown in panels (A) and (B), which is further overlaid on the CT image. In panel (C), longitudinal profiles, and in panel (D), lateral profiles whose locations are indicated in panel (A) with red dash lines are shown. Additionally, we add a homogenous magnetic field of 1.5T perpendicular to the transverse direction for each case to validate our magnetic field implementation. In Figure 8, the calculated doses in TOPAS (panel (A)) and ARCHER (panel (B)) are displayed. In panels (C) and (B), the profiles obtained from TOPAS without magnetic field are drawn in green lines as well. It can be observed that there are significant changes in dose distribution when the magnetic field was applied. TOPAS's results and ARCHER's results are still in good agreement.

To quantify the dose difference, 3D γ‐test are applied and results are shown in Table 2. The term “RS” indicates whether range shifters are applied in the treatment plan. In the 2 mm/2% criterion, mean γ‐test results for the original plan and magnetic plan are 99.7% and 99.3% respectively. To demonstrate the efficiency of our code, the computation time of ten million proton histories is listed in the last column of Table 2. The computation time of ARCHER ranges from 3.08 to 5.50 s. While TOPAS takes about 6000s running on a computation platform with the Intel(R) Xeon(R) CPU E5‐4607(48 threads). It's unfair to compare the computation time directly since they run on different computation platforms. Nonetheless, these numbers strongly demonstrate that our code is sufficiently efficient to be used in the clinical workflow.

**TABLE 2:** Quantitative analysis table for clinical patient cases.

Case	Energy (MeV)	$\bar{\sigma /D}$ (%)	RS	Original plan $P_{\gamma (2\%/2mm)}$ (%)	Magnetic plan $P_{\gamma (2\%/2mm)}$ (%)	T_ARCHER_ (s)	T_TOPAS_ (s)
Head‐and‐neck	139−170	0.59	Y	99.9	99.5	2.7	5286
	132−160	0.23	Y	99.8	99.5	2.4	4954
	99−127	0.79	N	99.5	99.2	2.3	4603
	99−115	0.86	N	99.6	99.3	2.3	4509
Breast	102−156	0.84	Y	99.5	99.0	2.9	5892
	102−159	0.91	Y	99.4	99.1	3.1	6256
	102−158	0.64	Y	99.7	99.3	2.9	5944
	102−150	0.91	Y	99.4	99.2	2.8	5897
Lung	102−191	0.54	Y	99.4	99.0	3.9	7879
	102−177	0.89	Y	99.6	99.1	3.6	7368
	102−184	0.84	Y	99.8	99.2	3.5	6901
Prostate	127−187	0.91	N	99.9	99.7	3.0	5774
	127−179	0.87	N	99.9	99.7	2.9	5941
	146−192	0.86	N	99.8	99.5	3.3	5477
	144−194	0.84	N	99.8	99.6	3.3	6348

## 4 DISCUSSION AND CONCLUSIONS

We have successfully developed a fast and accurate GPU‐based MC code for proton therapy. Proton transport follows the standard class II condensed history scheme with a continuous slowing down approximation. Nuclear interactions, ionization, energy straggling, and multiple scattering are modeled based on physics laws and/or reliable tabulated data. Moreover, protons deflected by the Lorentz force in magnetic fields are also implemented. To the best of our knowledge, no such GPU‐based MC code capable of performing proton dose calculations in magnetic fields has been reported. ARCHER is the first code that supports proton transport in magnetic fields and it can be applied to future MR‐guided protons therapy. Simulations in various tests indicate that the results produced with ARCHER are in good agreement with the TOPAS's results. Difference comparison metrics are used to quantify the agreement. The γ‐test passing rate with a 2 mm/2% criterion is over 99.2% for all cases. Even with a stricter criterion of 1 mm/1%, the minimum value of the γ‐test passing rate is 95.6%, clearly demonstrating the high accuracy achieved by ARCHER.

Using a single NVIDIA TITAN V card, the calculation time of 10^7^ source protons ranges from 0.82 to 5.50 s. Compared to TOPAS, the fast performance of our code can be attributed to two main advantages. The first advantage of our code is that the physical model implemented has been simplified effectively but without sacrificing accuracy, and the second is that our code programming strategy takes full advantage of GPU thread‐level parallelism and efficiency. The mean computational speed for all patient cases is 3.84 million protons/second in ARCHER, which is 0.5 million protons/second on an NVIDIA GTX680 card in Tseung et al.,^14^ 0.24 million protons/second on an NVIDIA TESLA C2075 card in Jia et al.,^12^ 0.29 million protons/second on four NVIDIA GTX980 cards in Schiavi ta al.,^13^ and 8.4 million protons/second on 1 or 2 NVIDIA RTX6000 cards in Fracchiolla et al.^15^ It should be noted that the computation speed strongly depends on the GPU device, voxel size, source energy, and phantom complexity. Nonetheless, our code is very competitive.

There are limitations to our current implementation. We found that in low‐density voxels, our dose is usually higher than TOPAS's. Similar problems have been identified in previous research. In our physics model, $T_{cut}$ is a constant (0.1 MeV) for all materials. However, in TOPAS, the user sets a range cut which is converted into difference $T_{cut}$ for different materials according to stopping power. $T_{cut}$ in low‐density materials, especially in the air, is usually much smaller than 0.1 MeV. This will lead to a larger restricted stopping power in our model and then cause higher dose. Additionally, the energy of all charged secondary particles except for protons is locally deposited. The range of secondary particles in low‐density materials is larger than the voxel size. Depositing all their energy will lead to an overestimation of the dose. To address these issues, we plan to consider $T_{cut}$ as a variable for different materials in future implementations. Furthermore, we intend to include electron transport in future code improvements. In fact, we have reported our success with electron and photon transport in GPU‐based code a decade ago.

To enhance code efficiency, the fictitious interaction method could be implemented in our code. In the current version, the cross‐section of three hard events must be calculated in each step, resulting in time‐consuming computations. To calculate the nuclear interaction cross‐section, each element's contribution must be accumulated, leading to an increase in computation time. In Table 1, we can see that the computation time of tissue is longer than that of water, although the tissue's stopping power is stronger, which means it tends to lose energy more quickly. The main reason is that tissue is composed of nine elements and it cost much more time to calculate the cross‐section. We propose the use of a fictitious interaction method, which employs a constant as the total cross‐section. This constant is derived from the summation of all hard event cross‐sections and a fictitious cross‐section. The fictitious cross‐section corresponds to the fictitious interaction, which is considered a hard event. Calculating the cross‐section of each hard event is only necessary when protons must go through a hard event. The proton remains unchanged if a fictitious interaction is sampled. Employing this method, we can further reduce computation time.

In applications, our code can be applied to patient‐specific third‐party quality assurance (QA) for secondary dose verification for radiation treatment plans exported from a commercial treatment planning system. It can realize automatic dose re‐calculation for clinical use, including data preparation, dose calculation, and 3D gamma analysis, and provide fast and accurate third‐party verification in order to discover errors and ensure patient safety. Additionally, our code can be adopted as the dose computation engine in the GPU‐based intensity‐modulated proton therapy (IMPT) optimization system. Currently, we only score the total dose and the required memory is equal to the number of voxels in the scoring grid(about 100 MB). But in an optimization system, one needs to score dose‐influence matrix, and the required memory is equal to the number of voxels in the scoring grid times the number of beamlets(about 100GB), which is much larger than GPU memory. Fortunately, Lee et al^37^ proposed an efficient data structure method, which only scores the voxels interacting with particles, to solve this problem. Furthermore, our code takes into account magnetic field effects on beam transport and dose distortions of proton beams, so it can be applied to future MRI‐guided proton therapy.

## AUTHOR CONTRIBUTIONS

Paper idea: Shijun Li, Xi Pei, and Xie George Xu. Physics Model: Shijun Li. Code implementation: Shijun Li and Bo Cheng. Validation: Shijun Li and Yuxin Wang. Writing of the paper: Shijun Li, Bo Cheng, Yuxin Wang, Xi Pei, and Xie George Xu.

## CONFLICT OF INTEREST STATEMENT

The authors declare no conflicts of interest.
